# Structural and dynamical insight into thermally induced functional inactivation of firefly luciferase

**DOI:** 10.1371/journal.pone.0180667

**Published:** 2017-07-03

**Authors:** Fatemeh S. Jazayeri, Mehriar Amininasab, Saman Hosseinkhani

**Affiliations:** 1 Department of Cell and Molecular Biology, School of Biology, College of Science, University of Tehran, Tehran, IRAN; 2 Department of Biochemistry, Faculty of Biological Sciences, Tarbiat Modares University, Tehran, IRAN; Russian Academy of Medical Sciences, RUSSIAN FEDERATION

## Abstract

Luciferase is the key component of light production in bioluminescence process. Extensive and advantageous application of this enzyme in biotechnology is restricted due to its low thermal stability. Here we report the effect of heating up above T_m_ on the structure and dynamical properties of luciferase enzyme compared to temperature at 298 K. In this way we demonstrate that the number of hydrogen bonds between N- and C-domain is increased for the free enzyme at 325 K. Increased inter domain hydrogen bonds by three at 325 K suggests that inter domain contact is strengthened. The appearance of simultaneous strong salt bridge and hydrogen bond between K529 and D422 and increased existence probability between R533 and E389 could mechanistically explain stronger contact between N- and C-domain. Mutagenesis studies demonstrated the importance of K529 and D422 experimentally. Also the significant reduction in SASA for experimentally important residues K529, D422 and T343 which are involved in active site region was observed. Principle component analysis (PCA) in our study shows that the dynamical behavior of the enzyme is changed upon heating up which mainly originated from the change of motion modes and associated extent of those motions with respect to 298 K. These findings could explain why heating up of the enzyme or thermal fluctuation of protein conformation reduces luciferase activity in course of time as a possible mechanism of thermal functional inactivation. According to these results we proposed two strategies to improve thermal stability of functional luciferase.

## Introduction

The process of light emission through chemical reaction in living organisms is called bioluminescence. Bioluminescent organisms are a diverse group including bacteria, fungi, algae, fish, squid, shrimp, and insects [1]. Luciferase is a general name of the enzymes that catalyze the oxidative reaction of light production. Luciferase structure, its substrate and cofactors varies in these organisms and it seems the bioluminescent systems have developed independently from different evolutionary origins [2].

Firefly luciferase is one of the best characterized and most applicable luciferase due to its distinct properties such as high quantum yield, high catalytic efficiency and substrate specificity [3–7]. Firefly luciferase requires ATP and O_2_ to catalyze a two-step oxidation of luciferin and generates light, oxyluciferin, CO_2_, and AMP [8]. North American *Photinus pyralis* luciferase (P08659 and EC 1.13.12.7), the first purified and characterized firefly luciferase is a 62 KD protein with 550 amino acids in mature form [9, 10]. Crystal structure of this enzyme has been determined at 2.0 Å resolution shown that protein consists of two compact domains separated by a wide cleft; a large N-terminal domain comprising a distorted antiparallel β-barrel and two β-sheets that are assembled to form a five-layered αβαβα structure and a small C-terminal domain with α+β architecture [11].

The wide requirements of labeling and monitoring in modern biotechnology and biological investigations and in order to achieve the proper capabilities of bioluminescence in these areas, enormous attention focused to the firefly luciferase and it has been largely used as a sensitive tool for continuous monitoring of biological processes such as gene expression, protein–protein interaction, cell death and apoptosis, tumor growth and metastasis in whole animals. Genetic reporter assays in molecular biology, clinical applications of drug screening and miniaturized bioanalytical devices such as microarrays and microfluidic devices are other applications of this enzyme [12–16].

Even with great successes in the usage of bioluminescence, there is still a broad field of study to improve performance of the system, such as developing functional characteristics for increasing luciferase stability. Since luciferase has low stability in 30°C, many groups focus on the study of the factors that enhance the luciferase stability. In this way, traditional techniques such as random mutagenesis [17, 18], rational design of mutations on the basis of the 3D structure, e.g. the mutagenesis of solvent exposed residues [19], introduction of disulfide bonds [20], and the comparative analysis [21] were applied. In other investigations, a collection of the old favored mutations simultaneously were used for construction of multi-point mutants [22, 23].

As mentioned above, the majority of previous works about luciferase stability were based on experimental methods using the crystal structure of enzyme as a model. One matter in these approaches is that the dynamic nature of atoms in the structure and its impact on the properties of the molecule are disregarded. Since proteins have structures with dynamical properties, the function of them is highly correlated with their intrinsic flexibility besides their structure. The dynamic of a protein allows its conformation to respond to the presence of other molecules and to changes of environmental factors. Biological and biochemical processes such as signal transduction, antigen recognition, protein transport and enzyme catalysis are based on this ability [24, 25].

Thus a fundamental description of how proteins work requires an understanding of the correlation between three-dimensional structures (traditionally solved by x-ray crystallography and NMR) and dynamic which is much more difficult to probe experimentally. Molecular dynamic simulation, the science of simulating of the motions of particles in a system according to the proposed force field, provides a useful bridge between structure and dynamic. The consequence of a molecular dynamic simulation is the solution of the time dependent equations of motion of particles using forces which are calculated from proposed interaction potentials to determine the dynamic of particles [26, 27].

There are still questions as to how protein dynamic and its flexibility affect its stability. Molecular dynamic simulation is an operative method that currently used for studying the thermostability of proteins affected by flexibility and atomic motions. Moreover, there are multiple examples of successful applications of MD methods to design thermostable proteins [28–31].

In this study, molecular dynamic was utilized to simulate free luciferase in explicit solvent at normal and at temperature above its T_m_. Ultimately the results of simulations were analyzed in order to evaluate the structural and dynamical properties of the enzyme.

## Results

### Homology modeling

The structural model of luciferase in free enzyme form was built by comparative homology modeling, S1 Fig. For free enzyme modeling three incomplete reported crystal structure of *Photinus pyralis* luciferase, with PDB codes 1BA3, 1LCI and 3IEP, were used as templates. 1BA3 has both N- and C-terminal domains and lacks residues between S198 and S201, a region in N-terminal domain and in the interface between two domains. Similar to 1BA3, 1LCI has both domains but lacks these residue segments: S198-L204, G355-P359, V435-I442, P523-L530. 3IEP has only N-terminal domain and in this domain it lacks residues between S199 and L204; D356 and P359. RMSD criterion was used to evaluate the structural similarity between model and templates. According to the DOPE score, S2 Fig, the best free model generated by Modeller among 100 trails had 0.08 nm RMSD with respect to the proposed templates. PROCHECK analysis for the quality of the constructed model revealed that 99% of residues were in the conformational allowed regions. The output of PROCHECH program for Ramachandran plot is depicted in S3 Fig. According to PROCHECK analysis of free of luciferase, two residues in disallowed region of Ramachandran plot are probably originated from the crystal pressure in original crystal structures and we expect to refine them in MD simulations.

### Molecular dynamic

Prepared model was subjected to 100 ns MD simulations under conditions as mentioned in methods section. The sampled trajectories of simulations in production stage were subjected to several analyses to characterize the structure and dynamic of luciferase and the impact of elevation of temperature on enzyme. The following abbreviations are assigned to our simulations; f298L (simulation of free luciferase at 298 K) and f325L (free luciferase at 325 K). In order to check whether the sampling of conformational space is sufficient, the production MD period was divided into four 25 ns parts and the cosine content of the first three principal components were calculated for each subtrajectory. The best results of cosine content were obtained for the last quarter part of the 100 ns simulations. The values of the first three principal components for the last time window were 0.47, 0.0, 0.0 for f298L and 0.12, 0.04, 0.01 for f325L.

#### Radius of gyration

As the indicator of the hydrodynamic size, the radius of gyration of enzyme was calculated along the trajectories and no significant differences were observed between f298L and f325L, with average value of 2.54±0.03 nm and 2.56±0.03 nm respectively.

#### Packing efficiency

The van der Waals volumes and Voronoi volumes over the simulation time were computed for N-, C-domain and complete protein structures. Voronoi volumes were calculated using the computational tool of trjVoroni [32]. Then the packing efficiencies of enzyme models as the ration of van der Waals to Voronoi volumes were calculated for two simulations along the trajectories. The comparison of the average values of packing efficiency showed no significant differences among two simulations.

#### Root Mean Square Deviation (RMSD)

**Fig 1:**
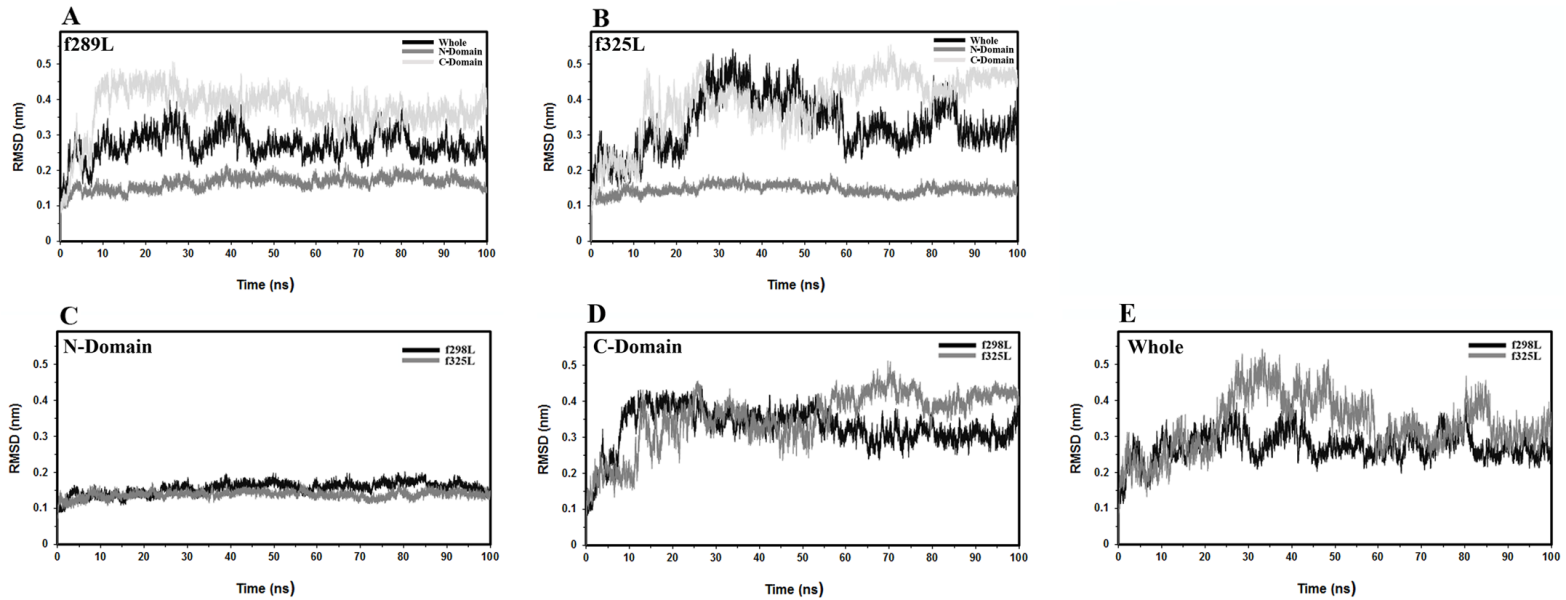
Time relaxation of enzyme structure obtained by backbone RMSD analysis. The Comparative diagram for various calculations of Backbone RMSD by regarding N-domain, C-domain and the whole protein as the reference fitting structure for (A) f298 and (B) f325. Comparisons between two simulations RMSDs calculated relative to (C) N-domain (D) C-domain and (E) whole protein backbone.

**Fig 2:**
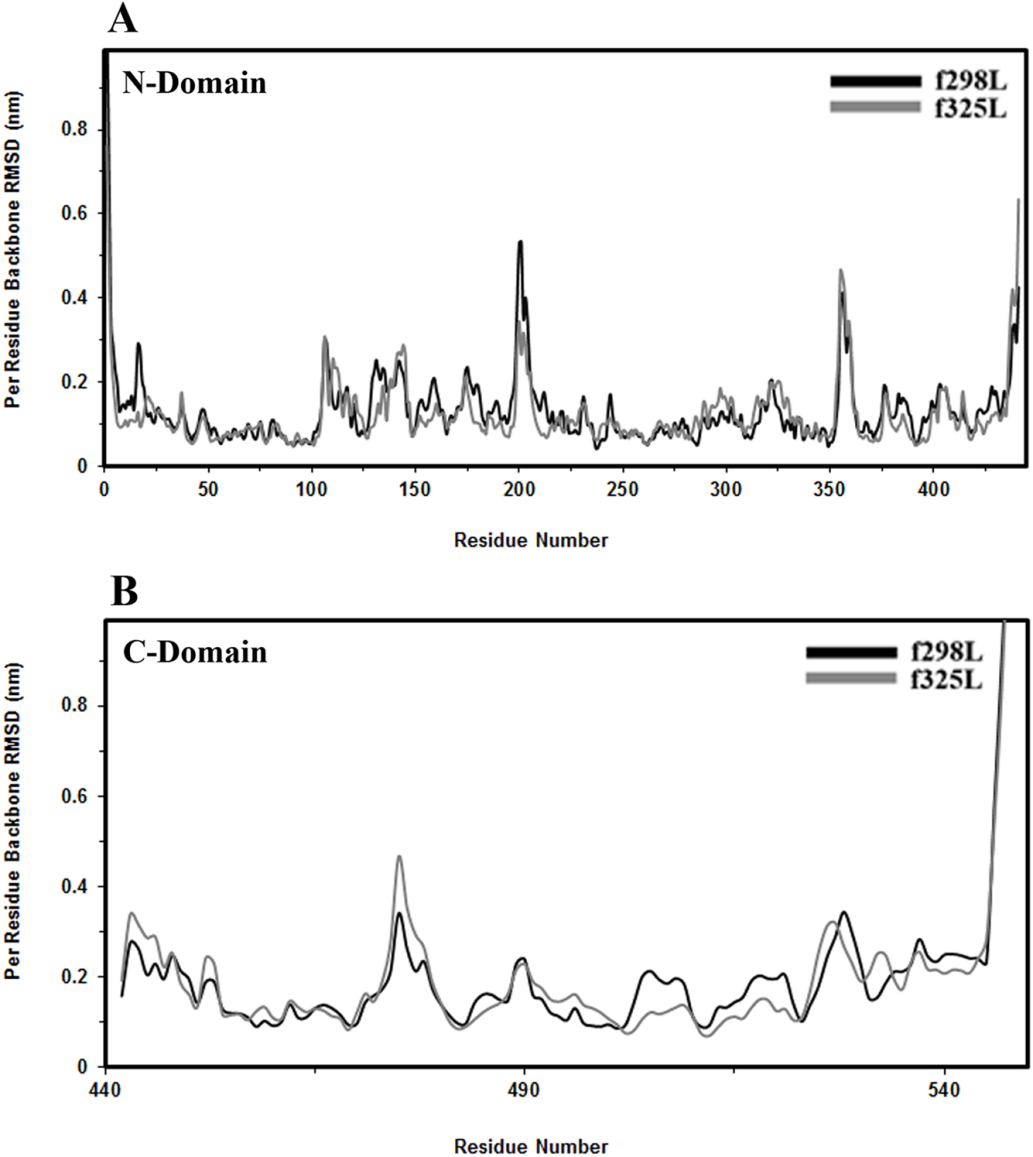
Per residue RMSD. Conformational changes within each domain based on per residue backbone RMSD calculated for two simulations by fitting to the backbone of corresponding domain (A) N-domain (B) C-domain.

The time evolution and relaxation of enzyme structure was monitored through the RMSD calculation. The RMSD values of the best fitting of backbone atoms of each frame with respect to its own initial structure were calculated as a function of time for all proposed simulations by regarding N-domain, C-domain and the whole protein as the reference point, Fig 1. The corresponding average values of RMSD along the trajectories were summarized in S1 Table. According to the whole protein structure, f325L undergoes more conformational deviation relative to its own initial structure with respect to f298L behavior. By regarding the deviations of individual domains, the conformational variation of N-domain for f325L shows a little bit less deviation relative to its own initial N-domain relative to f298L. On the contrary f325L shows more deviation by considering only the C-domain. Therefore it could be concluded that the C-domain undergoes more conformational changes along with possible reorientation of N- and C-domain relative to its own initial conformation in course of simulation trajectory. Furthermore the heating up the free leuciferase from 298 K to 325 K shows more deviation as a whole. In order to investigate the conformational changes within each domain, the average deviation of each residue with respect to its own initial domain structure was calculated for all tree simulations by fitting to secondary structure elements of corresponding domain, Fig 2. Analysis of RMSD per residue was done separately and comparatively. For all simulations, the largest value of RMSD was related to residues of floppy tails of the molecule. With regard to other residues, some regions with relatively higher values of RMSD can be recognized in each domain as follows: 1- The amino acid range S199-L204 within N-domain located in the loop connecting strands A6 and A7 of β-sheet A. This loop is exposed to the solvent in the large cleft separating the N-terminal and C-terminal domains and belonging to the highly conserved sequence motif [STG]198-[STG]-G-[ST]-[TSE]-[GS]-X-[PALIVM]-K206. Maximum deviation in this region occurred to f298L. 2- The amino acid range G355-G360 within N-domain which is a part of the loop connecting strand B8 to strand C2. f298L and f325L showed maximum deviation in this region among the simulations. 3- The amino acid range D475-D476 within C-domain, a part of the exposed turn connecting strands E1 and E2. f325L showed maximum deviation in this turn. 4- The amino acids range K525-K529 within C-domain which located in loop connecting strand E3 and helix 15. This region contains many conserved residues among homologous enzymes and like the second region is exposed to the solvent and located in the large cleft separating the N-terminal and C-terminal domains. Maximum deviation in this region occurred to f325L. In addition to the conformational changes in each domain, the linker region connecting N- and C-domain was analyzed. The amino acids between D436 and Q448, part of it acts as a linker (D436-S440) between N- and C-domain, showed conformational changes. Interestingly, structural change in this region changed the relative orientation of N- and C-domain with respect to each other. f325L showed major changes in relative orientation of N- and C-domain which reflects the structural relaxation of free enzyme in solution by heating from 298 K to 325 K. In contrast, f298L showed minor changes and retained its original relative orientation.

#### Cluster analysis

In order to investigate the conformational diversity of each simulation the cluster analysis was performed for the last 25 ns part of trajectories using various values of RMSD cutoff, S2 Table. As S2 Table shows, the heating up of the enzyme increased the variety of available conformational states, as expected.

#### Secondary structure analysis

The secondary structure of each residue for two simulations was assigned as a function of time using DSSP program, S4 Fig. Additional information about the content of each secondary structure element was gained via the analysis of secondary structure map produced by DSSP program [33]. As S3 Table reveals, the average content of each secondary structure element shows minor variations among two simulations.

#### Root Mean Square Fluctuation (RMSF) per residue

**Fig 3:**
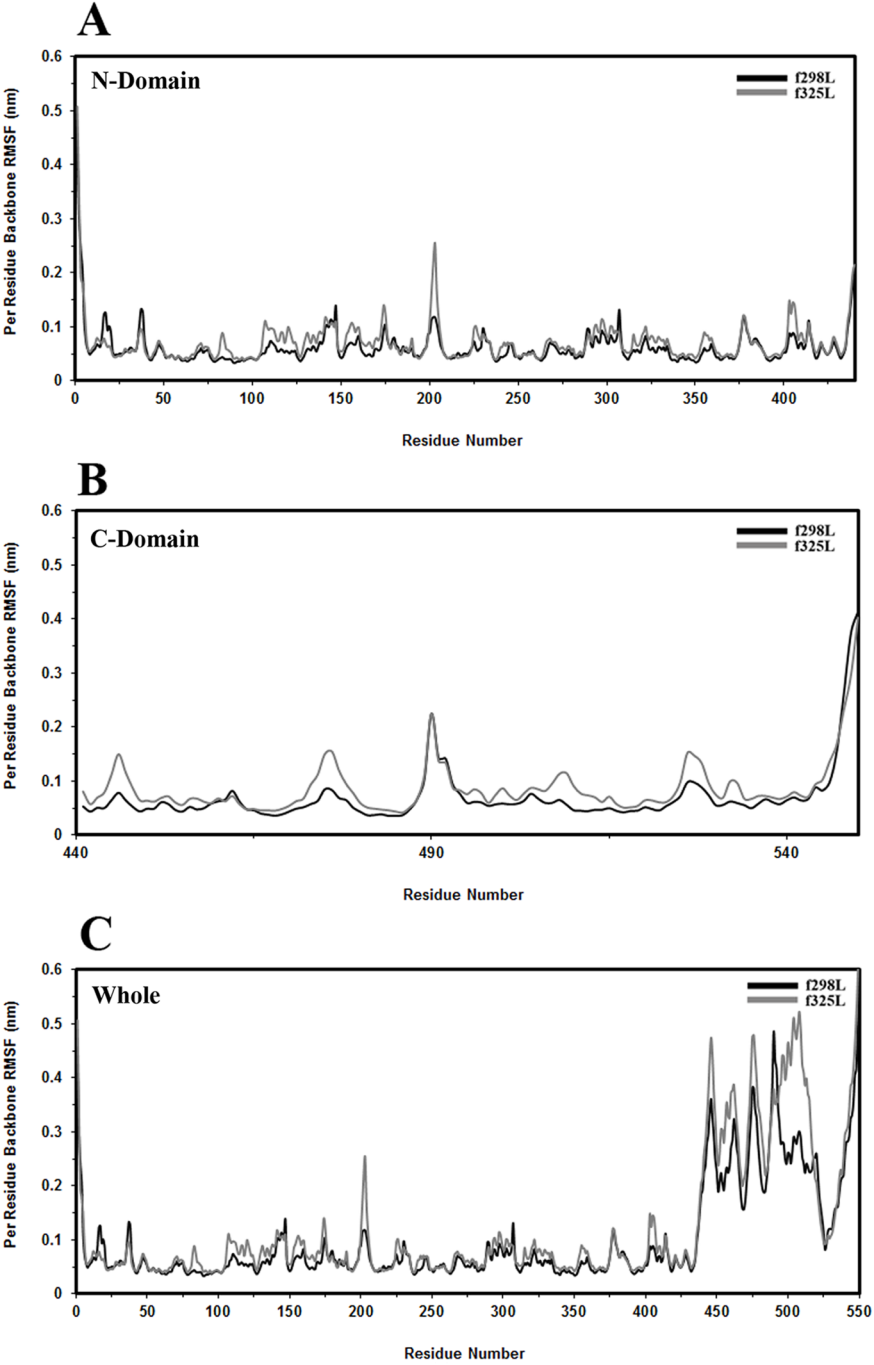
RMSF. The structural flexibility of simulated models (f298L and f325L). (A) Per residue RMSF of N-domain by fitting to the N-domain of reference structure (B) Per residue RMSF of the C-domain by fitting to C-domain of reference structure (C) Per residue RMSF of the whole protein by fitting to N-domain of reference structure.

RMSF is a criterion for amino acids flexibility that measures the average deviation of the center of each residue or residue part with respect to its mean position. In order to explore intra and relative inter domain motions, for the last 25 ns part of each trajectory, the per residue RMSF values of the backbone atoms were calculated in three following situations: 1- Per residue RMSF of the N-domain by fitting to N-domain of reference structure, Fig 3A; 2- Per residue RMSF of C-domain by fitting to C-domain of reference structure, Fig 3B; 3- Per residue RMSF of the whole protein by fitting to N-domain of reference structure, Fig 3C. By considering the absolute value of per residue RMSF and the ratio of f325L per residue RMSF to corresponding values of f298L, the flexible regions in each simulation were explored.

As Fig 3A shows, elevation of temperature from 298 K to 325 K caused increased flexibility in P173-G175 loop which connecting helix 6 to strand A6 and G200-P205 sequence region. Amino acid range G200-P205 within N-domain is located in the loop connecting strands A6 and A7 of β-sheet A. This loop as mentioned in RMSD section, is exposed to the solvent in the large cleft separating the N-terminal and C-terminal domains and belonging to the highly conserved sequence motif [STG]198-[STG]-G-[ST]-[TSE]-[GS]-X-[PALIVM]-K206. In both of f298L and f325L simulations, the linker region (D436-S440) has higher flexibility.

The heating up of the enzyme causes the elevation of flexibility in K445-Y447, D474-A477 and G525-G528 (the loop at interface of domains) amino acid, Fig 3B. It seems the linker segment and region centered at G446 play an import role in enzyme flexibility and function since as Fig 3C shows the simultaneous flexibility of the linker and K445-Y447 regions cause the collective motion of C-domain relative to N-domain which could be important in expression of enzyme activity. As Fig 3C shows, this flexibility combination expresses itself in collective motion of C-domain relative to N-domain in both of f298L and f325L simulations with a larger extent of motion in f325L.

#### Hydrogen bond analysis

Hydrogen bond is one of the most important molecular interactions that affects protein folding and structure and specificity of protein interactions with other proteins and ligands. Therefore the study of hydrogen bonding pattern and its alterations in different situations could be informative.

At first the average number of hydrogen bonds in each time frame was calculated for the last 25 ns of proposed simulations, Table 1. By considering f298L as the reference point, a significant reduction in total hydrogen bonds occurs in f325L. The contribution of intra and inter domain hydrogen bonds also was calculated. The same reduction trend was observed for intra domain hydrogen bonds with respect to f298L. Interestingly, an average increase of three in inter domain hydrogen bond numbers was occurred to f325L with respect to f298L. This observation implies that the heating up of the enzyme strengthens the inter domain contacts of two domains. This strengthen by heating up could mechanistically explain the functional time decay of the enzyme activity.

**Table 1:** Hydrogen bond statistics.

H-bond number	Total	Within N-domain	Within C-domain	Between N- and C-domain
**f298L**	**394.6 ± 8.7**	**318.8 ± 7.6**	**69.3 ± 4.2**	**6.6 ± 1.4**
**f325L**	**390.7 ± 9.7**	**316.0 ± 8.6**	**65.3 ± 4.2**	**9.5 ± 1.7**

The statistics of the number of hydrogen bonds in the last 25 ns of each simulation.

S5 Fig shows the existence maps of all two simulations over entire simulation time. Analysis of the percentage of existence probability for residues involved in hydrogen bonds was done on the last 25 ns of each simulation to resolve intra and inter domain contributions.

The number of hydrogen bonds with existence percentage change greater than 40% and 75% were determined (S4 Table) and identified. S5 Table presents the identity and the existence probability percent for those donor-acceptor pairs of f325L in comparison to f298L which show greater than 75% changes for N- and C-domain. The similar results are shown in S6 Table for inter domain hydrogen bonds with changes greater than 40%. As these tables show, the heating up could change the three dimensional hydrogen bond pattern of the enzyme, which correspondingly modifies the structural and dynamical properties. As S6 Table shows, the appearance of two simultaneous strong salt bridge and hydrogen bond between K529 and D422, and R533 and E389 in f325L could mechanistically explain stronger contact between N- and C-domain. According to the reported data in S6 Table for f325L, the overall effect of increasing the existence percentage and the appearance of new hydrogen bonds between two domains, strengthen the inter domain contact and modulate their relative orientation. The change in hydrogen bond pattern for f325L is related to the contact between the loop connecting strand E3 and helix 15 from C-domain and strand C5 and strand C4 from N-domain.

#### Active site region

Luciferase like other members of large family of adenylating enzymes catalyzes a two-step reaction; in the first step the enzyme adenylates luciferin and in the next step the oxidative decarboxylation of luciferyl-AMP intermediate occurs to produce light. Crystallographic and modeling methods along with mutagenesis studies revealed a detailed picture of substrate binding site of luciferase. Conserved residues that are responsible for substrate binding and catalysis reaction are located on the interface of two domains in the large cavity of enzyme. Two domains in an open structure form a closed structure upon substrate binding to embrace the substrate which its benzothiazole ring is tightly sandwiched in a hydrophobic pocket. Moreover it has been shown that after the first step of catalysis reaction the rotation of C-domain occurs and enzyme adopts a new conformation which capable to catalyze the second step [34].

Residues that locate in binding site of enzyme have been determined on the basis of crystal structures. In addition, P loop (phosphate binding loop) is a universal motif in ATP binding enzymes and comprises the signature sequence of luciferase superfamily [35] and it is supposed that this loop has an important role in substrate binding of firefly luciferase (S198-K206). Another loop (also called active site loop [34] with residue range K524-L530) from C-domain which includes functional K529 along with the P loop make the entrance region to substrate binding site and probably have a vital role in substrate binding and enzyme reactivity.

To investigate the impact of temperature rising on substrate binding and enzyme activity we have compared the solvent accessible surface area (SASA) of active site residues with respect to f298L, Table 2. As it is shown in Table 2, total SASA of active site residue is reduced from 5.21 nm^2^ for f298L to 4.33 nm^2^ for f325L. The functional role of two active site residues, T343 and K529, in adenylation step has been proved by mutational studies [36–38] which show notable reduction of SASA for f325L with respect to f298L. Interestingly as mentioned above, the side chain of K529 make simultaneously a strong salt bridge and hydrogen bond with carboxylic group of D422 in f325L as shown in Table 2. These findings, e.g. reduced solvent accessibilities, could explain why heating up of the protein or thermal fluctuation of protein conformation reduces enzyme activity in course of time.

**Table 2:** Solvent accessible surface area.

Active Site Residues	SASA (nm^2^) f298L	SASA (nm^2^) f325L
**HIS245**	**0.88**	**0.81**
**PHE247**	**0.49**	**0.49**
**THR251**	**0.28**	**0.23**
**GLY316**	**0.30**	**0.17**
**ALA317**	**0.31**	**0.26**
**GLN338**	**0.38**	**0.36**
**GLY339**	**0.02**	**0.02**
**TYR340**	**0.19**	**0.29**
**THR343**	**0.37**	**0.17**
**ALA348**	**0.24**	**0.22**
**ASP422**	**0.48**	**0.31**
**LYS529**	**1.27**	**1.00**
**SUM**	**5.21**	**4.33**

Comparison between f298L and f325L according to the calculated solvent accessible surface area (nm^2^) of the functionally important residues of active site.

#### PCA analysis

The effect of temperature rising on concerted motion of luciferase was investigated by PCA/ED analysis. The last 25 ns part of trajectories of two proposed simulations have been used to construct the covariance matrices after fitting to their own reference structure to remove overall protein translation and rotation. In order to simplify the analysis, only the alpha carbon positions of each trajectory were used.

**Fig 4:**
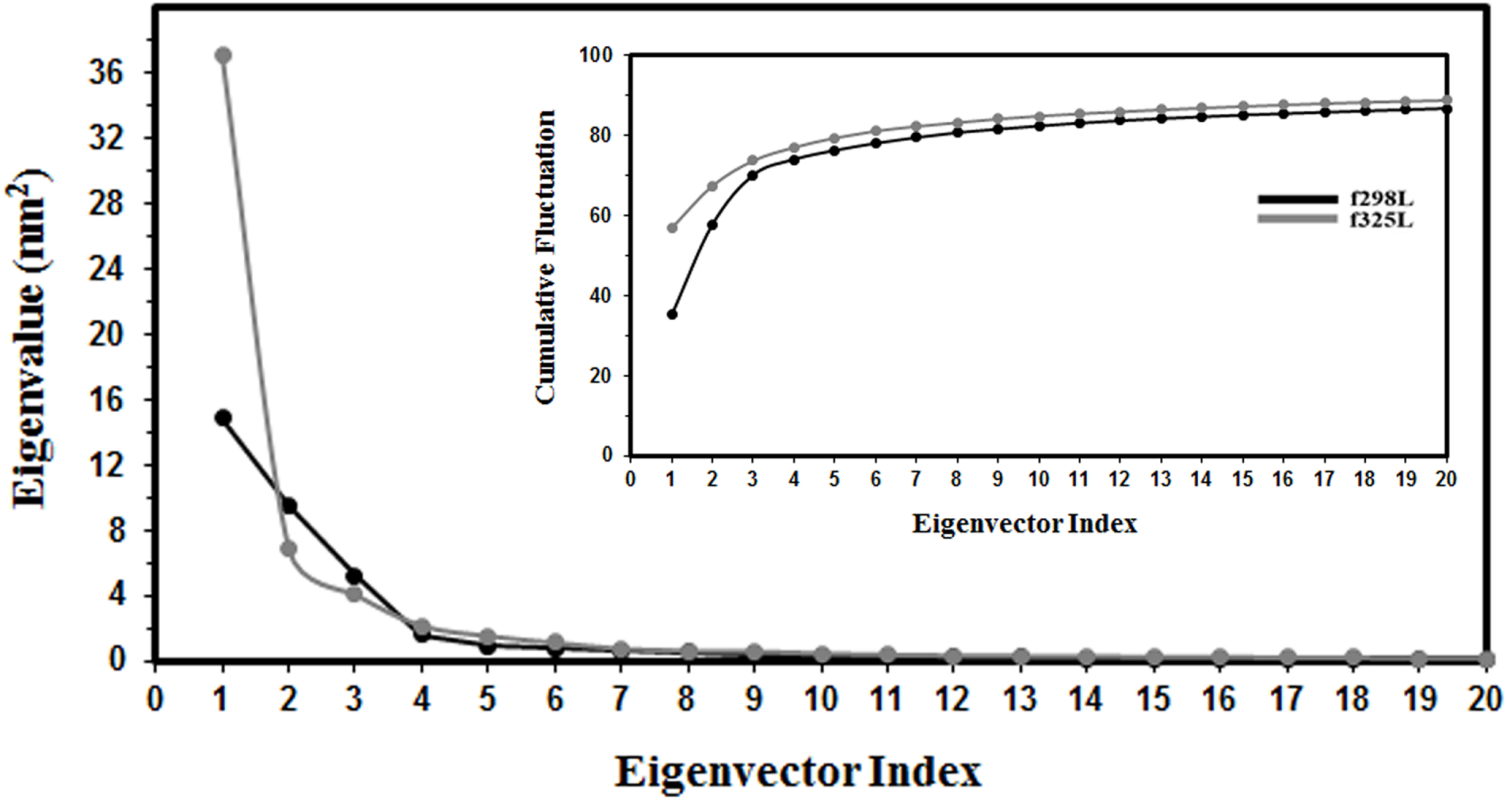
Eigenvalues. The eigenvalues of the first 20 eigenvectors in descending order for f298L and f325L MD simulations. The inset shows the cumulative contribution of eigenvectors.

Fig 4 shows the eigenvalues of two simulations in descending order and relative cumulative fluctuation against the corresponding eigenvector indices. The eigenvalue curve for f325L is steeper with respect to f298L. Since the eigenvalues are the average square displacement along their own eigenvectors and the first eigenvalue has the most contribution to the protein motion and as it is clear from Fig 4, f325L shows a 2.5 fold increase for the first eigenvalue relative to f298L. On the contrary the second eigenvalue of f325L shows a 1.5 fold decrease relative to corresponding value of f298L which implies a reduction in average displacement along the second eigenvector. Under the same force field, the reason for these differences could be related to the increasing the temperature and we can conclude that the motion modes and extent of motions are modulated by heating up of the enzyme. The third eigenvalue for f325L is a little bit less than the corresponding value for f298L. Other eigenvalues are the same for f325L and f298L. Also as Fig 4 shows, the first tree eigenvalues contribute to about 70 and 75 percent of motion for f298L and f325L respectively. These findings show that effective dimensionality of the internal motions of protein is restricted to decreased dimensions and less complexity of motion for f325L with respect to f298L upon rising the temperature.

**Fig 5:**
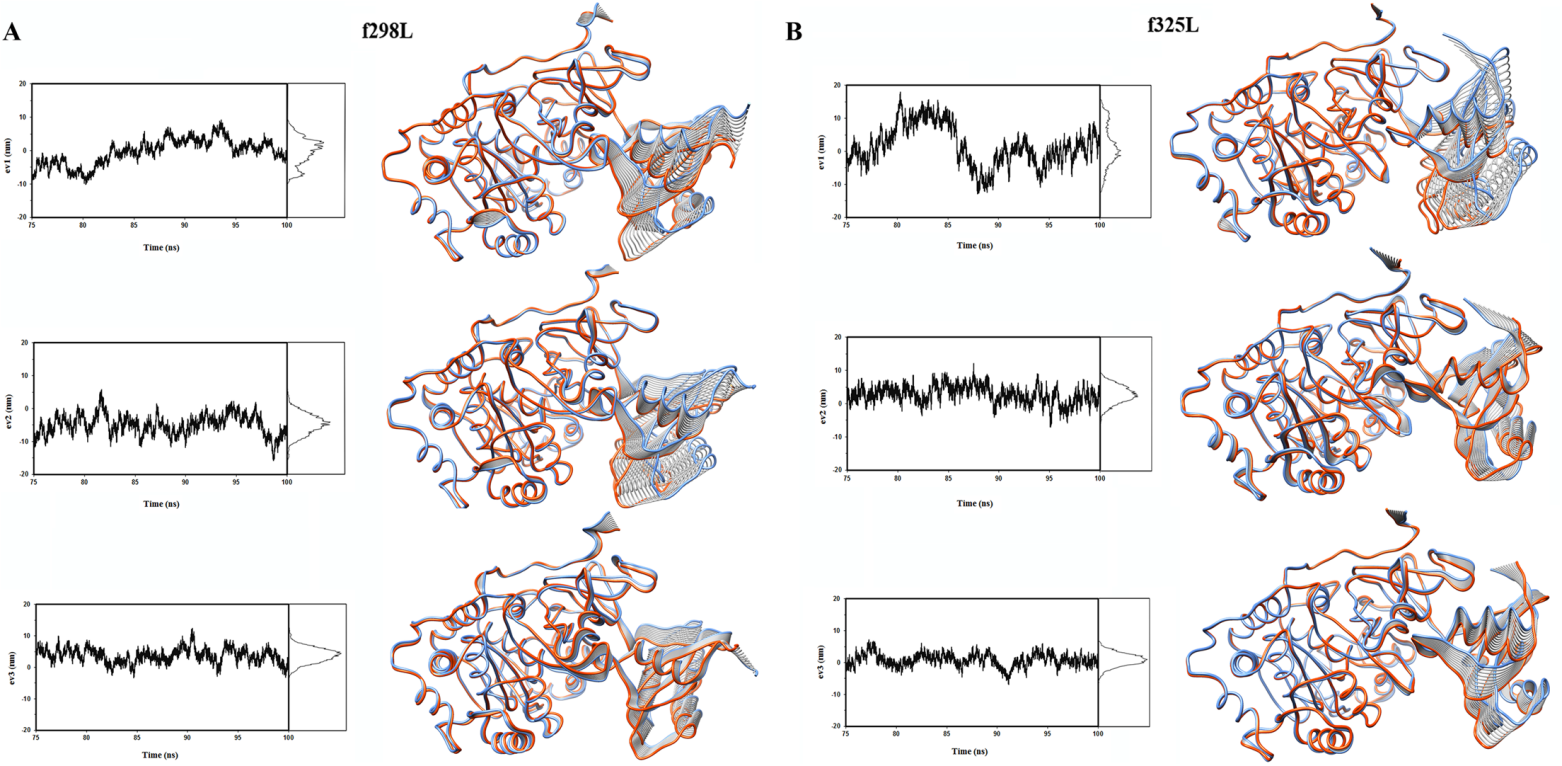
The projection of simulation trajectory on the first three eigenvectors and corresponding probability distributions. Also two extreme structures along with conformations of the linear interpolation between them are shown. (A) f298L. (B) f325L.

By projecting the trajectory onto each eigenvector one can have a closer look at eigenvalues and motions along important eigenvectors. Fig 5 shows the projection on the first three eigenvectors and corresponding probability distributions. Accordingly, at the right part of Fig 5A and 5B two extreme structures along with conformations of the linear interpolation between them are shown.

As Fig 5 and S1–S6 Animations shows, the main motions of protein are related to the reorientation motion of C-domain with respect to N-domain. Also Fig 5 and S1–S6 Animations reveals that upon temperature rising, dynamical changes of enzyme structure mainly originated from the change of motion modes and associated extent of those motions with respect to f298L.

**Fig 6:**
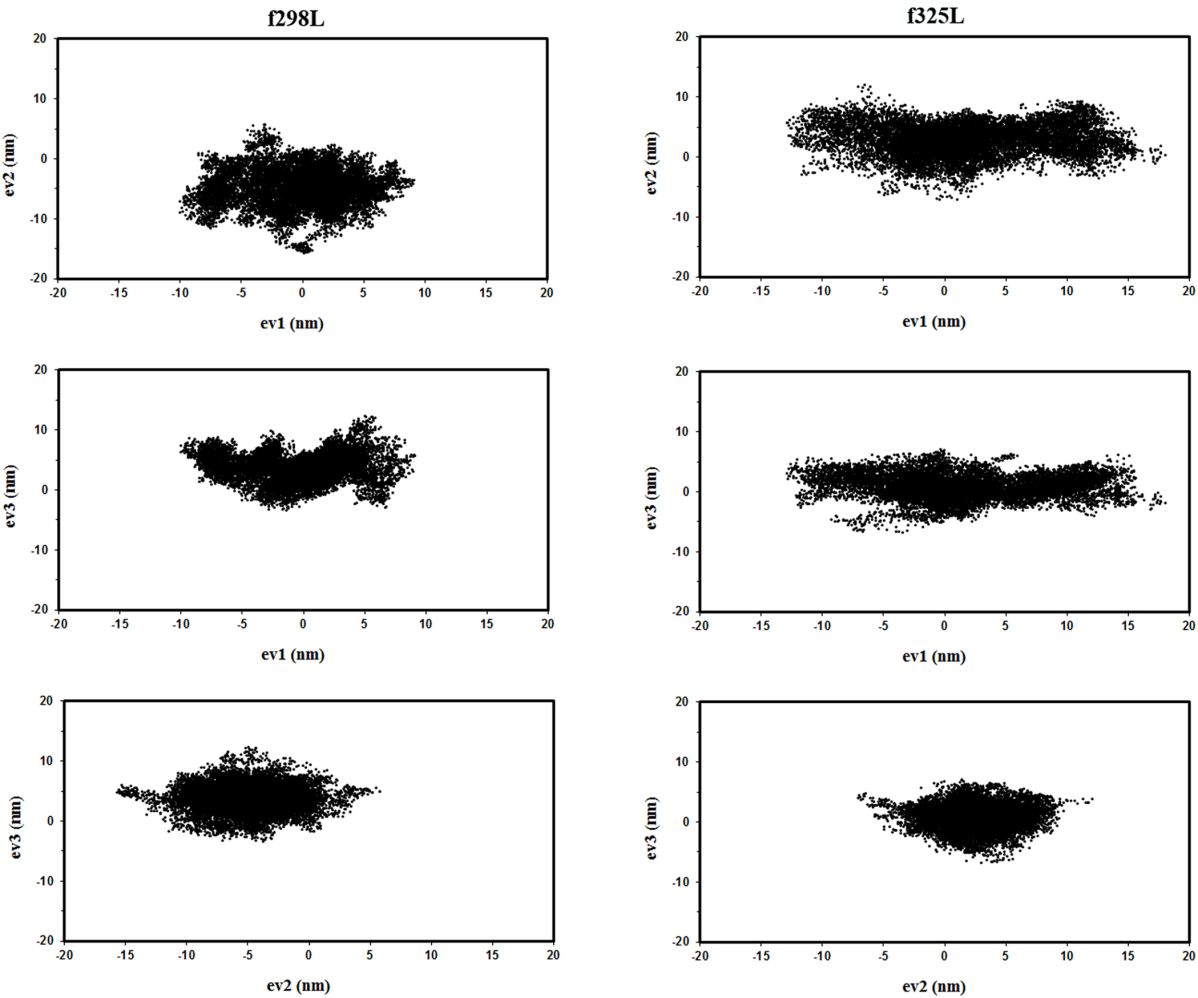
The projected trajectories. The projection of trajectories of f298L (left) and f325L (right) on planes defined by eigenvectors 1, 2; 1, 3 and 2, 3.

Fig 6 shows the projection of trajectories on planes defined by eigenvectors 1, 2; 1, 3 and 2, 3. Regarding f289L as the reference point, f325L shows displacement of trajectory region and the confinement of it in broader range especially for eigenvector 1 as expected by rising the temperature. This finding supports the higher flexibility of f325L at 325 K with respect to f298L mainly at linker and C-domain.

**Fig 7:**
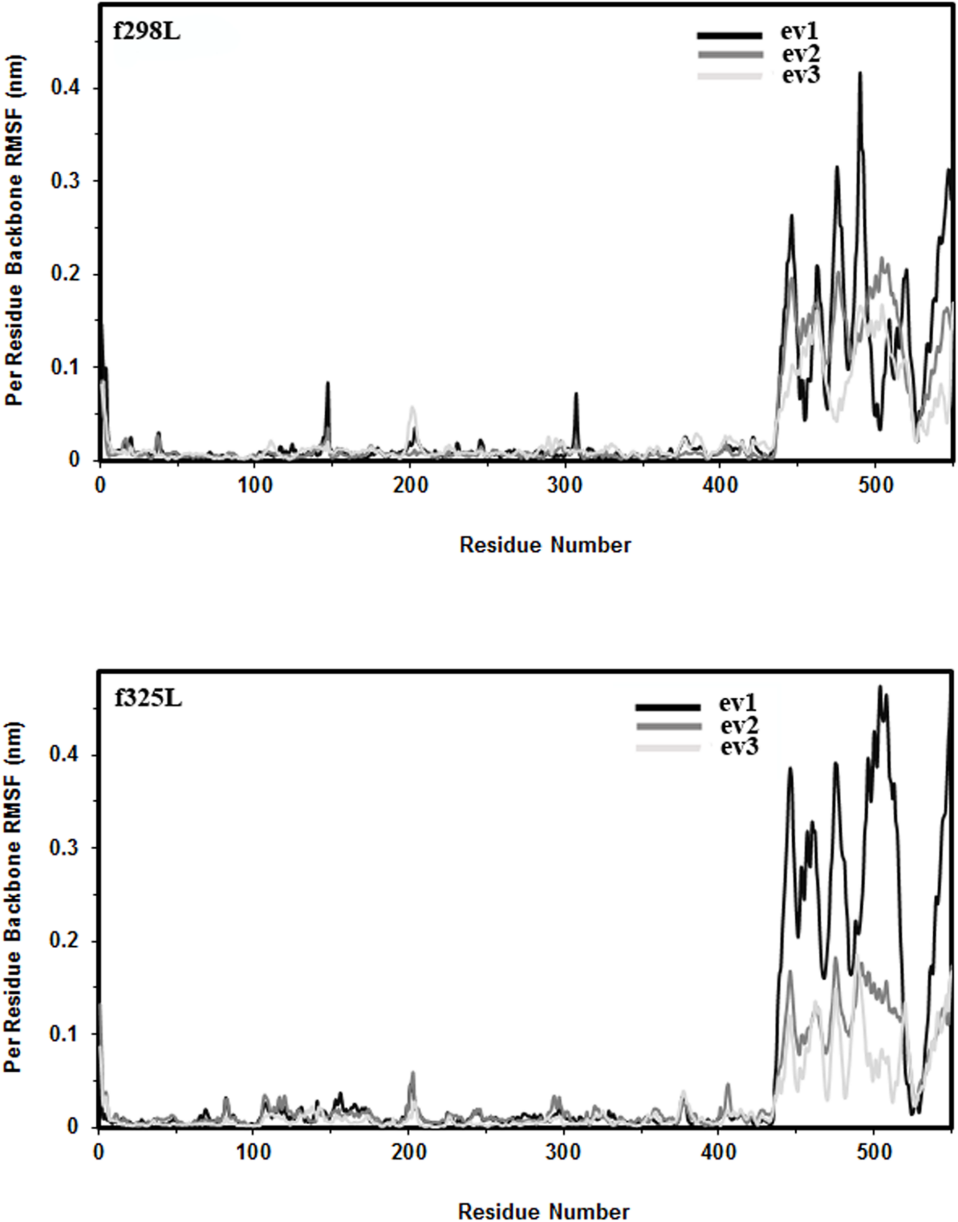
RMSF. Per residue backbone RMSF of the first three eigenvectors for f298L (top) and f325L (bottom) simulations.

Fig 7 shows the RMSF values of the first three eigenvectors for f289L and f325L simulations. In complete agreement with RMSF section, higher flexibility is observed in C-domain which mainly resulted from the collective motion relative to N-domain. As expected the eigenvector 1 shows higher contribution in protein flexibility for all two simulations. Considering f298L as the reference point, heating up the enzyme causes elevated flexibility of enzyme.

## Discussion

Experimental studies including random mutagenesis and rational design have been used extensively to explain and improve thermal stability and activity of firefly luciferase. These factors are the most serious problematic parameters which limit luciferase usage. In spite of many outcomes, the approach of these methods was based on crystal structure of the enzyme. In this regard, the dynamic nature of protein and its impact on enzyme behavior (structure dynamic relationship) is disregarded. The dynamic behavior of proteins is difficult to probe experimentally, but the molecular dynamic simulation provides an atomistic approach in this regard.

In this study, molecular dynamic simulation was applied to investigate dynamical behavior of firefly luciferase enzyme. In this way, the influence of temperature above the T_m_ on luciferase structure and dynamic was investigated in two separate 100 ns simulations.

Analysis of Radius of gyration and Packing efficiency confirm that molecular sizes and their fluctuations were nearly equal for f298L and f325L simulations which indicates overall folding of enzyme did not disturb after heating up. DSSP analysis shows minor variations in the content of each secondary structure element among two simulations upon heating up.

Inspection of conformational diversity through cluster analysis indicates that an increase in the variety of available conformational states occurred for f325L.

For the case of f325L simulation, i.e. in case of heating up of the enzyme, we can summarize other analyses as follows: 1- The RMSD data represent a notable reorientation of C-domain relative to N-domain in comparison with f298L. Per residues RMSD analysis shows conformational changes in the linker region connecting two domains beside changes in each domain. 2- Flexibility of protein was examined through RMSF analysis. Heating up of the enzyme causes increased flexibility in some regions such as G200-P205 and G525-G530 loops from two domains that both of them exposed to the solvent in the large cleft of enzyme and have a critical role in enzyme function. In addition, flexibility of linker region increases as a result of the elevation of temperature that expresses itself in relative motion of C-domain with respect to N-domain. 3- Despite of decreasing of the total number of hydrogen bonds, heating up increases inter domain hydrogen bonds with increments of three in average. Among them the appearance of simultaneous strong salt bridge and hydrogen bond between K529 and D422 is notable. Mutagenesis studies suggest that K529 is very important residue for effective substrate orientation and it provides favorable polar environment and interactions to stabilize the transition state which leading adenylate reaction proceeds efficiently [38]. Similar studies show that D422 is an essential residue for luciferase activity [11]. Also the existence probability of simultaneous strong salt bridge and hydrogen bond between R533 and E389 is significantly increased. Thus the overall effect of increasing the existence percentage or the appearance of new hydrogen bond between two domains, strengthen the inter domain contact and modulate their relative orientation. 5- Study of residues located in active site region revealed that solvent accessible surface area, SASA, of two important catalytic residues, i.e. K529 and T343 show notable decrease for f325L. A reduction of about 1 nm^2^ in SASA of active site residues along with simultaneous formation of hydrogen bond and salt bridge between K529 and D422 causes the active site becomes less accessible to enzyme substrate. These findings could explain why heating up of the protein or thermal fluctuation of conformation reduces enzyme activity in course of time.

PCA analysis for motion mode shows that effective dimensionality of the internal motions of protein is restricted to decreased dimensions and less complexity of motion for f325L with respect to f298L. Also the dynamical behavior of enzyme is changed upon heating up which mainly originated from the change of motion modes and associated extent of those motions with respect to f298L.

To improve thermal stability of functional luciferase, two strategies could be proposed. One approach is based on engineering of flexible residues by rigidifying them through RMSF analysis [39]. One critical point should be considered is, this manipulation should not affect the required flexibility for enzymatic reaction.

The other approach could be based upon the prevention of two simultaneous hydrogen bonds and salt bridges proposed in our study that strengthen the inter domain contact (D422/K529 and E389/R533). K529 belongs to A10 conserved motif and earlier studies investigated that it is critical in adenylation state of luciferase reaction and provides favorable polar interactions required for transition state stabilization. Mutations at K529 including K525A, K529Q and interestingly conservative substitution K529R show considerably lower specific activity [23,36–38,40,41]. Asp422 is one of six absolutely conserved residues in luciferse superfamily and is essential for proper positioning of AMP [42]. Therefore it seems that mutations at K529 or D422 could destruct enzyme activity.

R533 also located in A10 motif, but its role in enzyme catalysis have not been reported [41]. E389 placed near the active site region and make a hydrogen bond with G421, but there isn’t any reported data about mutations of this residue [42]. Thus it could be logical to consider the effect of R533 and E389 rational mutations on thermal stability and activity of luciferase enzyme.

## Materials and methods

### Homology modeling

The complete three dimensional structure of *Photinus pyralis* luciferase (P08659 and EC 1.13.12.7) in free form was built by comparative homology modeling as the initial structure for further analysis by molecular dynamic simulations. Modeller [43] was used for homology modeling to generate the 3D structure of each proposed model by satisfying spatial restraints imposed by the specified multiple sequence alignment. The sequence of *Photinus pyralis* mature luciferase (P08659) was fed to Basic Local Alignment Search Tool for Proteins (BLASTP) from National Center for Biotechnology Information (NCBI) to search against Protein Data Bank (PDB) by keeping its default algorithm parameters. To build the homology model of free luciferase structure, three incomplete structures of *Photinus pyralis* luciferase (1BA3, 1LCI and 3IEP PDB codes with 2.2, 2 and 2.1 Å resolution respectively) were taken as template structures and Modeller9v10 software simulation was used for model building. Modeller input alignment file was created manually via the alignment of three sequences produced by ClustalX program [44]. To guarantee sufficient conformational sampling, the generation of 100 models was supposed for satisfactory sampling. The selection of the best model was based on the DOPE assessment score. The best constructed model was then validated by PROCHECK [45] for the quality of structures.

### Molecular dynamic

In order to refine and analyze the structural and dynamical behavior of the generated models by homology modeling, molecular dynamic simulation method was applied. All molecular dynamic simulations were carried out with the GROMACS simulation package version 4.5.4 [46, 47] using the Amber99SB force field parameters [48] as implemented in GROMACS. The protonation state of the titratable groups of luciferase at pH 7.0 was determined after estimating their pK_a_ values by H++ server (accessible at http://biophysics.cs.vt.edu/H++). Form 13 existing histidines, H310 and H419 were considered to be positive and the others were supposed to be neutral. Two sets of simulations were performed using the generated models in homology modeling as the initial structure: free luciferase at 298 K and free luciferase at 325 K (above the T_m_ of enzyme). For each simulation the enzyme was centered in periodic cubic box with edges of about 10 nm. The boxes were solvated with pure water using TIP4P-Ew water molecules [49]. To achieve a neutral simulation box, the net charge of the protein was neutralized by replacing water molecules with necessary Na^+^ ions. In order to remove bad atomic contacts, each solvated and neutralized system was subjected to energy minimization using the steepest descent algorithm until the maximum force was smaller than 500 kJ/mol.nm. After energy minimization, two separate position-restrained MD simulations were carried out. First, to set the atomic velocities and adjust the system temperature, an MD simulation was performed for 100 ps at desired temperature and in a constant volume condition (NVT). Second, to adjust pressure and densities, an MD simulation with 1 ns duration was carried out at constant temperature and pressure (NPT). In all of the NPT simulations, temperature and pressure were kept close to the intended values (298 K or 325 K and 1 bar) by using the modified Berendsen thermostat (V-rescale) [50] and Berendsen barostat algorithm [51] with τ_T_ = 0.1 and τ_P_ = 0.5 ps respectively. The LINCS algorithm was utilized to constrain all bonds [52]. A single-range cutoff was applied for calculation of the non-bonded interactions. The cutoff radius was set to 1.2 nm for both Coulombic and Lennard-Jones interactions. The Coulombic interactions of longer range were calculated by means of the particle-mesh Ewald (PME) algorithm [53]. After equilibration, all two sets of production MD simulations were carried out for 100 ns duration. Time steps of 2 fs duration were used and frames were collected every 2 ps.

In order to evaluate the quality or sufficiency of conformational sampling of simulations, the production MD period was divided into four 25 ns parts and principal component analysis was performed on each sub-trajectory. Eigenvectors and eigenvalues were obtained from the diagonalization of the covariance matrices of the Ca atoms, and the principal components were generated by projecting the trajectories on the respective eigenvectors. The cosine content of the principal components was calculated to estimate whether the conformational fluctuations are connected with the potential (when the cosine content is close to 0) or with random diffusion (when the cosine content is close to 1) [54].

The collective and correlated motions of a protein can be detected through the analysis of molecular dynamic simulations via principal component analysis (PCA) or essential dynamic (ED) [55]. This is especially a useful method to compare the behavior of similar systems subjected to different conditions. The PCA or ED method is based on the construction of the covariance matrix of the coordinate fluctuations of CA atoms of enzyme along the trajectory after fitting to a suitable reference structure. After diagonalization of the covariance matrix, the information about the collective and correlated motion of protein was obtained from a few chosen eigenvectors and eigenvalues which account for the most variations in the system without much loss of information.

## Supporting information

S1 Fig 3D representation of constructed homology model of free firefly luciferase (PDB codes of templates: 1BA3, 1LCI and 3IEP; target model is in yellow color). (TIF)

S2 Fig DOPE score per residue profile of best model generated by MODELLER for free firefly luciferase. (TIF)

S3 Fig Ramachandran plot of best generated free model of firefly luciferase produced by PROCHECK program. Plots statistics are shown in number of residues and percent of residues.
(TIF)

S4 Fig Time dependent secondary structure variation of residues for f298L (A) and f325L (B) simulations using the DSSP program. Assigned color codes show the occurrence of each secondary structure element.
(TIF)

S5 Fig Hydrogen bond existence maps over entire simulation time for f298L (A) and f325L (B). Presence of a hydrogen bond in each time is indicated by red color.
(TIF)

S1 Table Average values of backbone RMSD over entire trajectory of each simulation. (TIF)

S2 Table Conformational diversity of each simulation in terms of the number of calculated clusters for different values of RMSD cutoff. (TIF)

S3 Table The percentage probability of each secondary structure element for f298L and f325L simulations. (TIF)

S4 Table The number of hydrogen bonds with existence percentage change greater than 40% and 75% for f325L simulation with respect to f298L. (TIF)

S5 Table The donor-acceptor pairs and the existence probability percent for f325L simulation which show greater than 75% changes in the existence probability for N- and C-domain in comparison to f298L. (TIF)

S6 Table The donor-acceptor pairs and the existence probability percent for f325L simulation which show greater than 40% changes in the existence probability for inter domain hydrogen bond in comparison to f298L. (TIF)

S1 Animation The motion along eigenvector 1 for f298L simulation. (MPG)

S2 Animation The motion along eigenvector 2 for f298L simulation. (MPG)

S3 Animation The motion along eigenvector 3 for f298L simulation. (MPG)

S4 Animation The motion along eigenvector 1 for f325L simulation. (MPG)

S5 Animation The motion along eigenvector 2 for f325L simulation. (MPG)

S6 Animation The motion along eigenvector 3 for f325L simulation. (MPG)
