# Supplementary figures and images for: Structural and dynamical insight into thermally induced functional inactivation of firefly luciferase
Source: PLoS One. 2017 Jul 3;12(7):e0180667. doi: 10.1371/journal.pone.0180667 (PMC5495494; doi:10.1371/journal.pone.0180667)

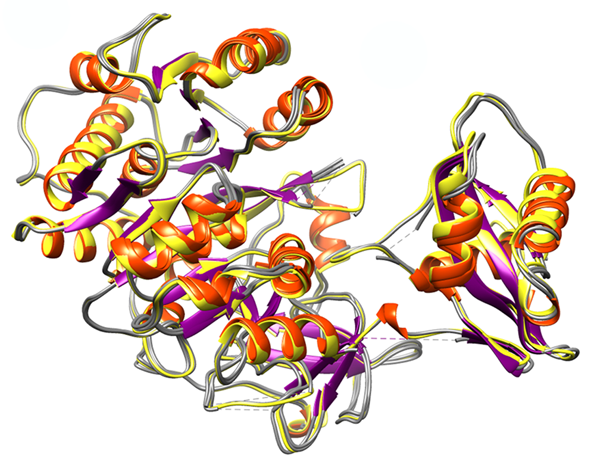

Supplement: S1 Fig — (TIF) [file pone.0180667.s001.tif]

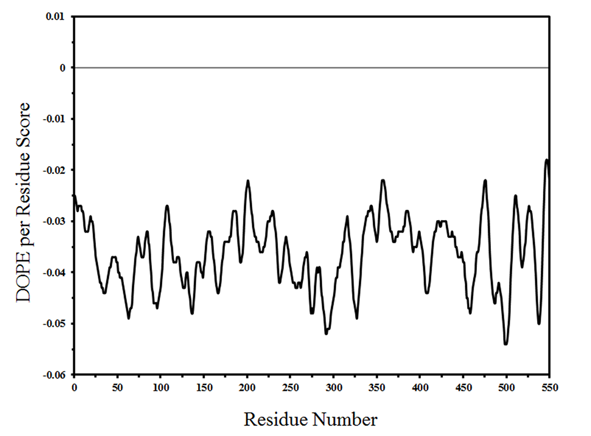

Supplement: S2 Fig — (TIF) [file pone.0180667.s002.tif]

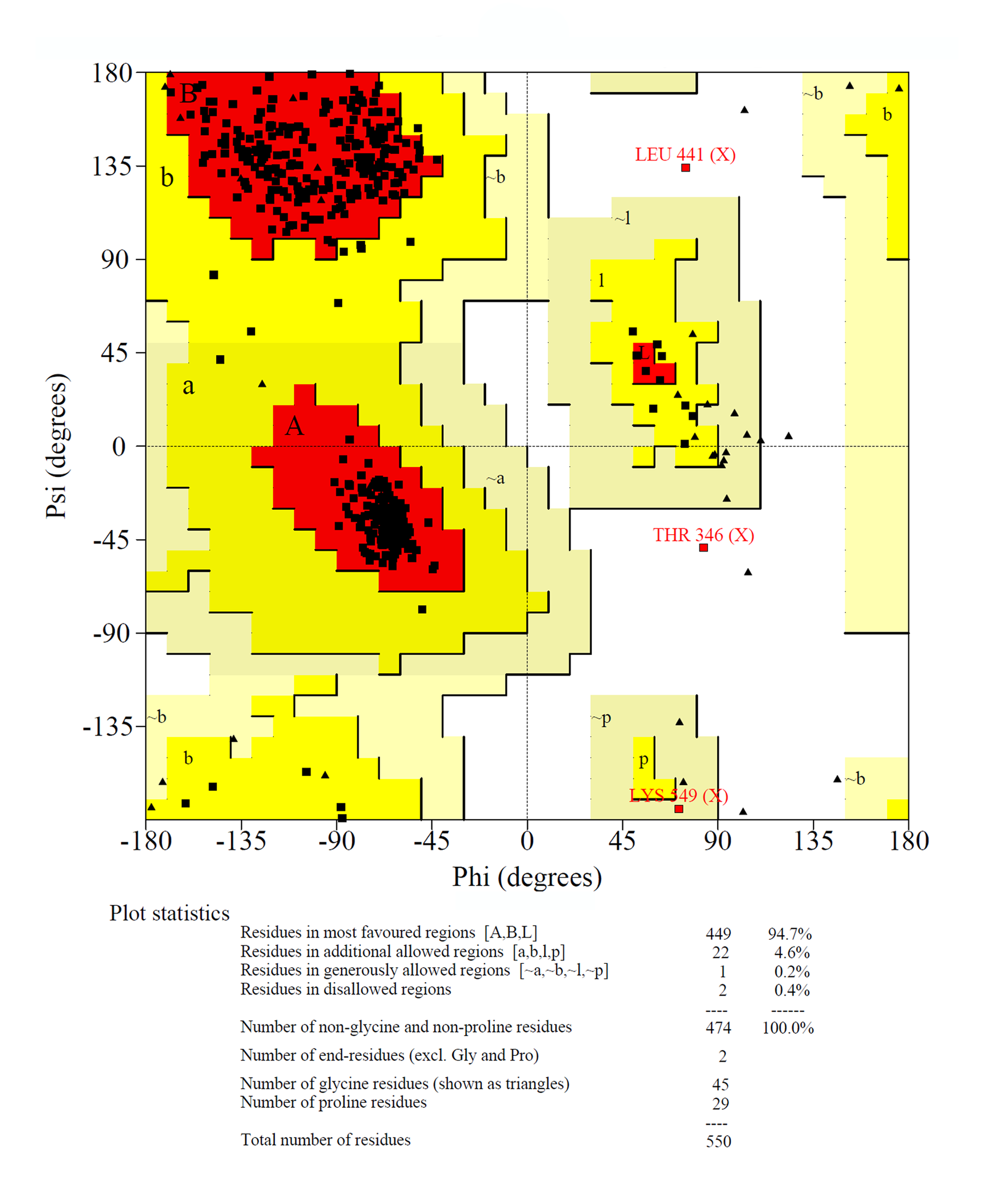

Supplement: S3 Fig — Plots statistics are shown in number of residues and percent of residues. (TIF) [file pone.0180667.s003.tif]

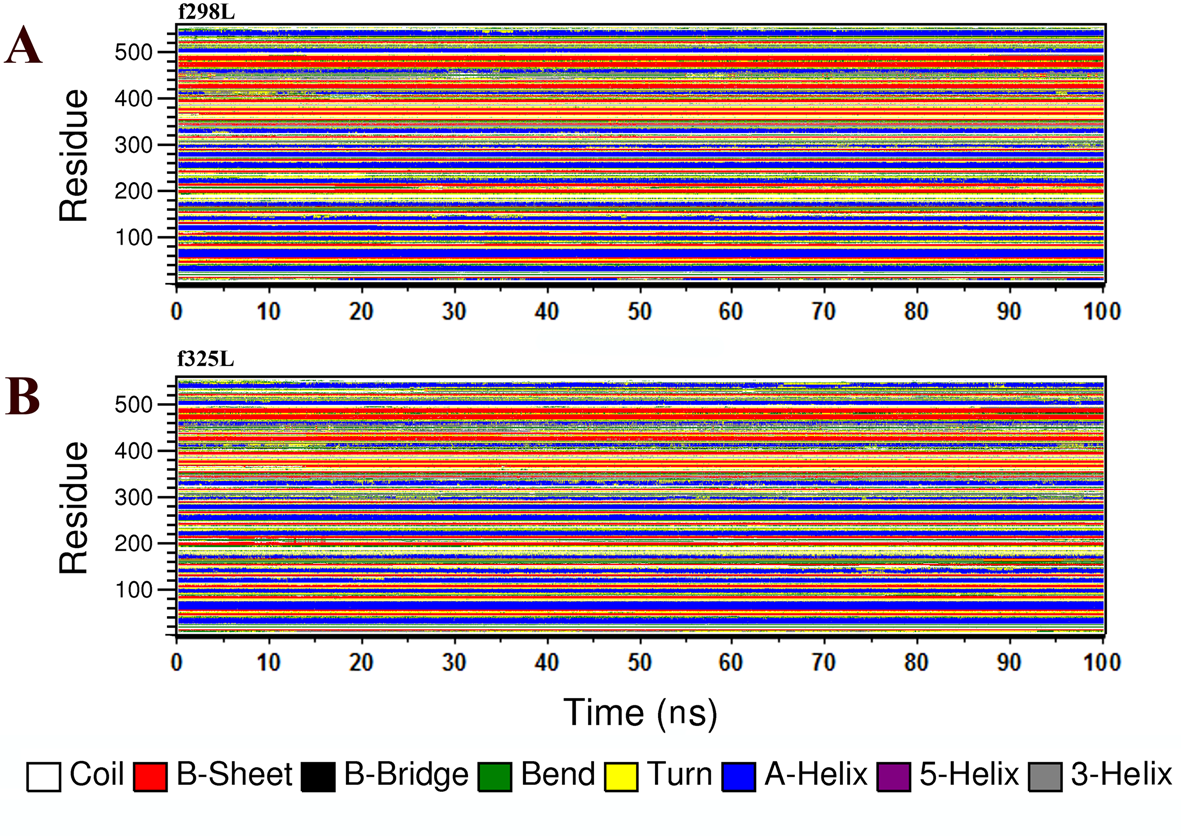

Supplement: S4 Fig — Time dependent secondary structure variation of residues for f298L (A) and f325L (B) simulations using the DSSP program. Assigned color codes show the occurrence of each secondary structure element. (TIF) [file pone.0180667.s004.tif]

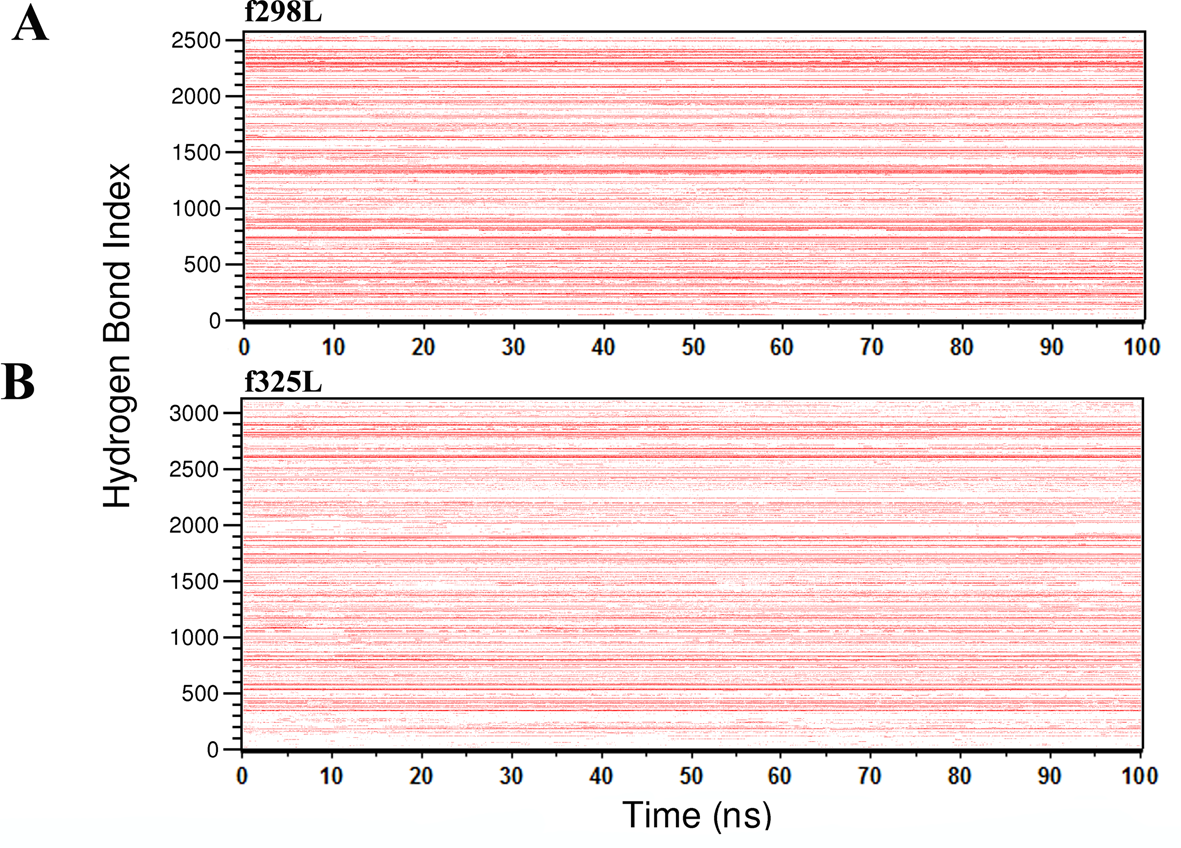

Supplement: S5 Fig — Hydrogen bond existence maps over entire simulation time for f298L (A) and f325L (B). Presence of a hydrogen bond in each time is indicated by red color. (TIF) [file pone.0180667.s005.tif]

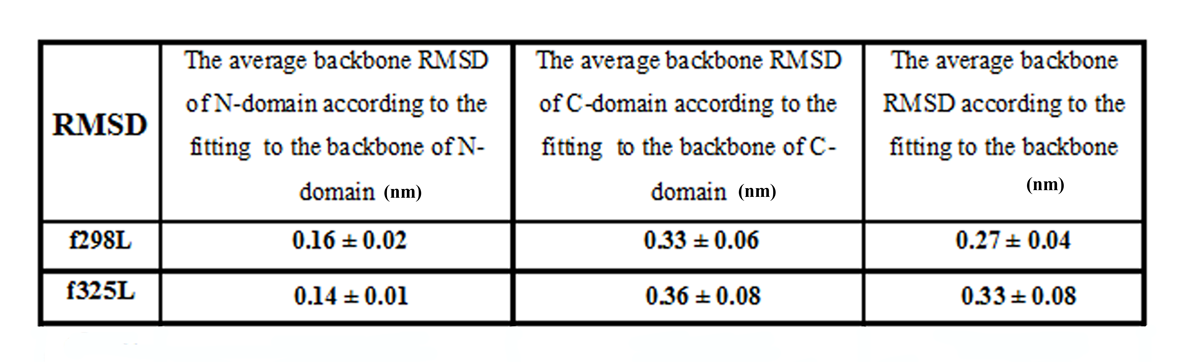

Supplement: S1 Table — (TIF) [file pone.0180667.s006.tif]

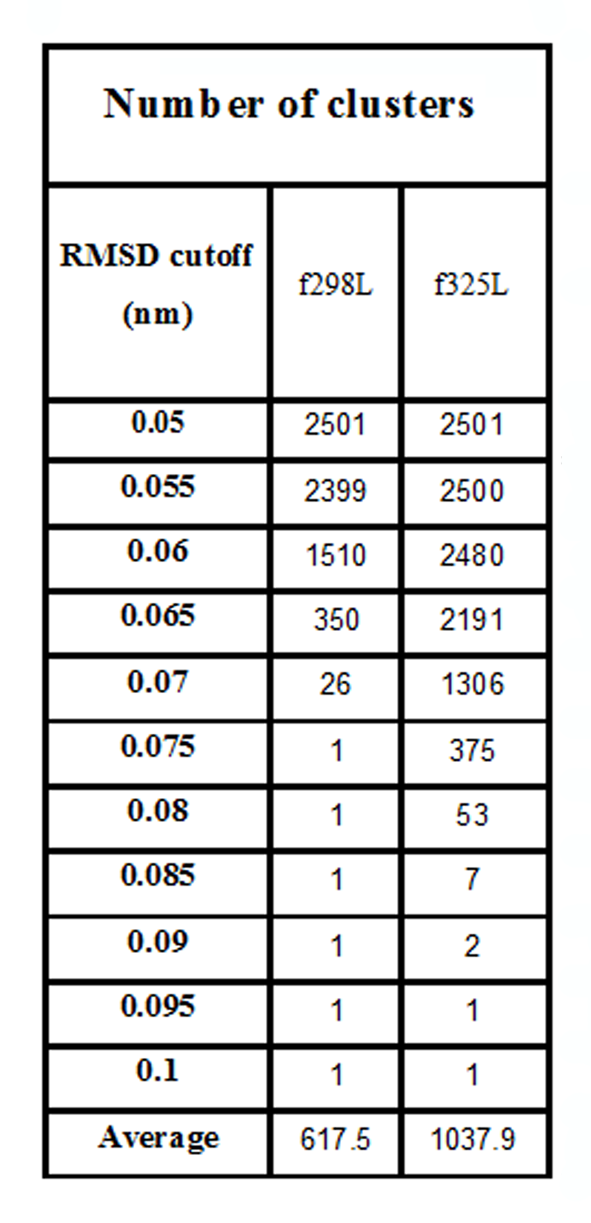

Supplement: S2 Table — (TIF) [file pone.0180667.s007.tif]

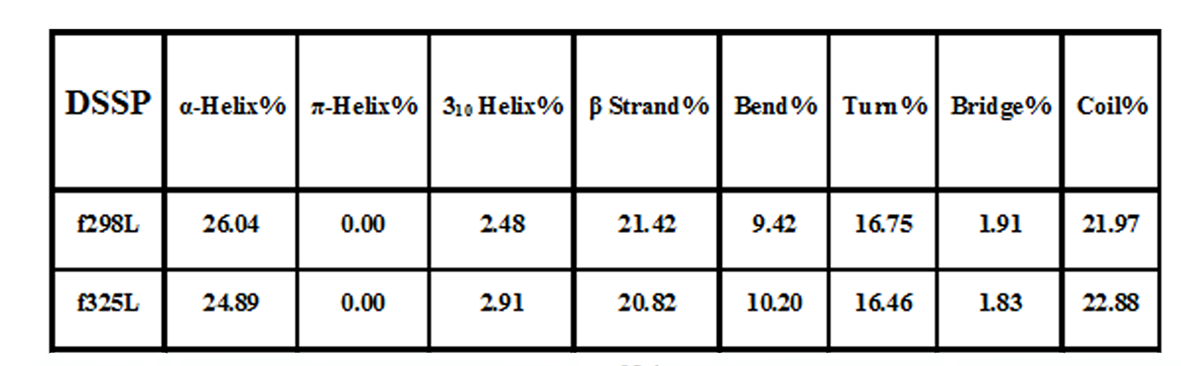

Supplement: S3 Table — (TIF) [file pone.0180667.s008.tif]

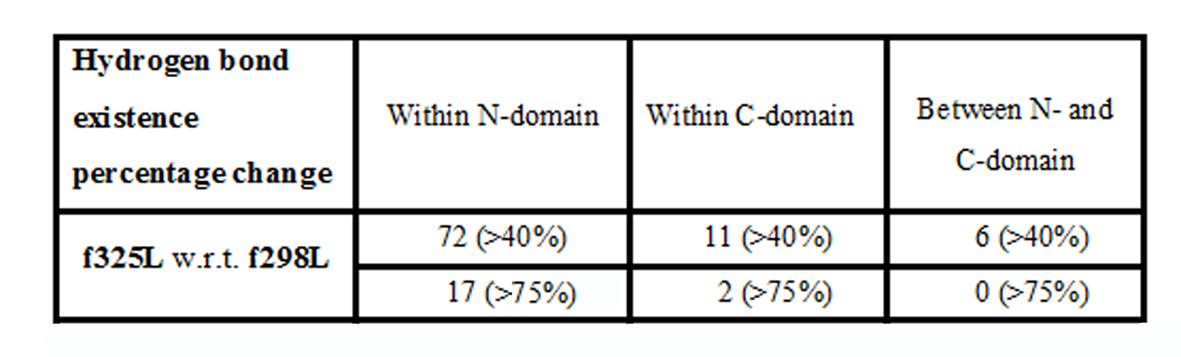

Supplement: S4 Table — (TIF) [file pone.0180667.s009.tif]

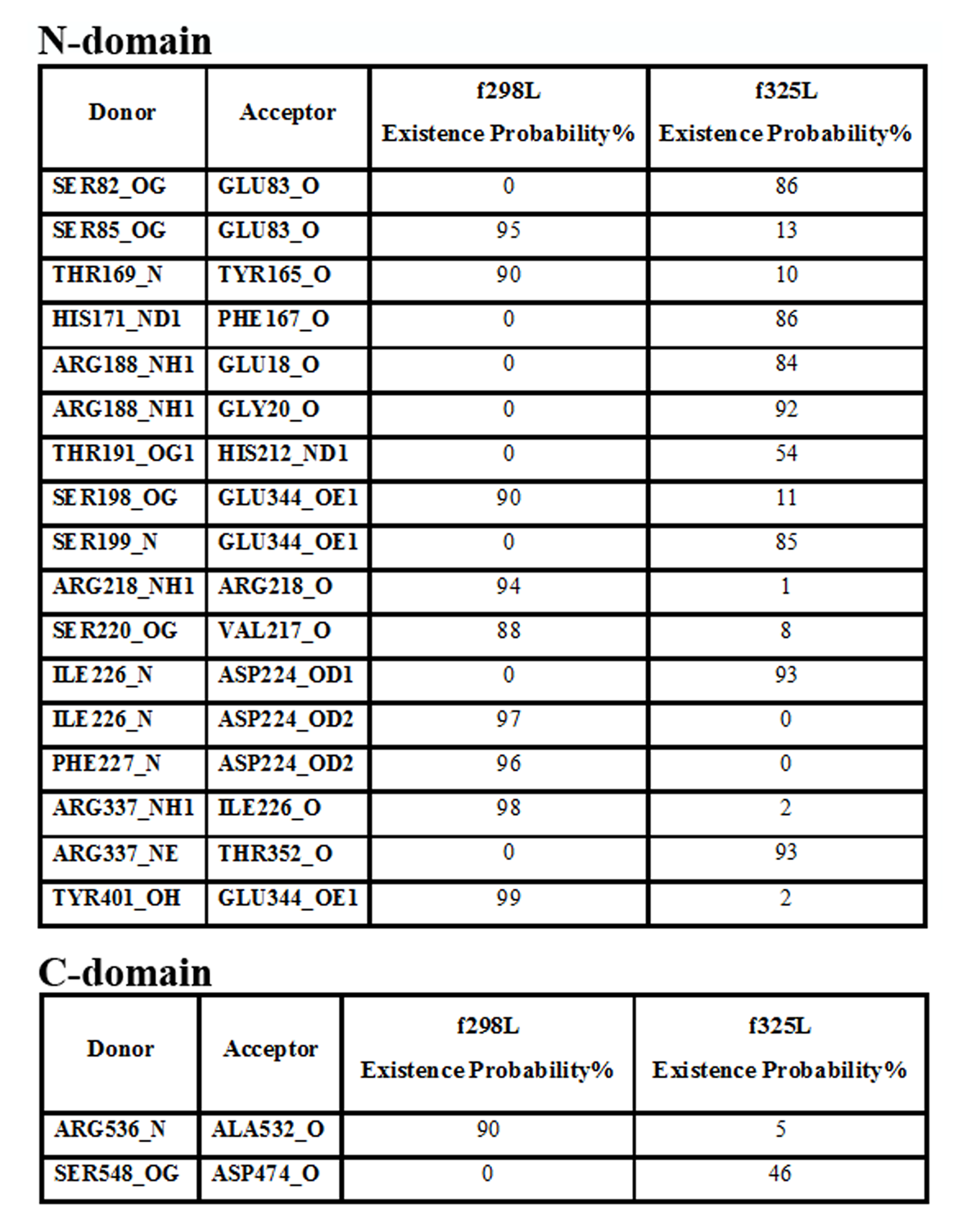

Supplement: S5 Table — (TIF) [file pone.0180667.s010.tif]

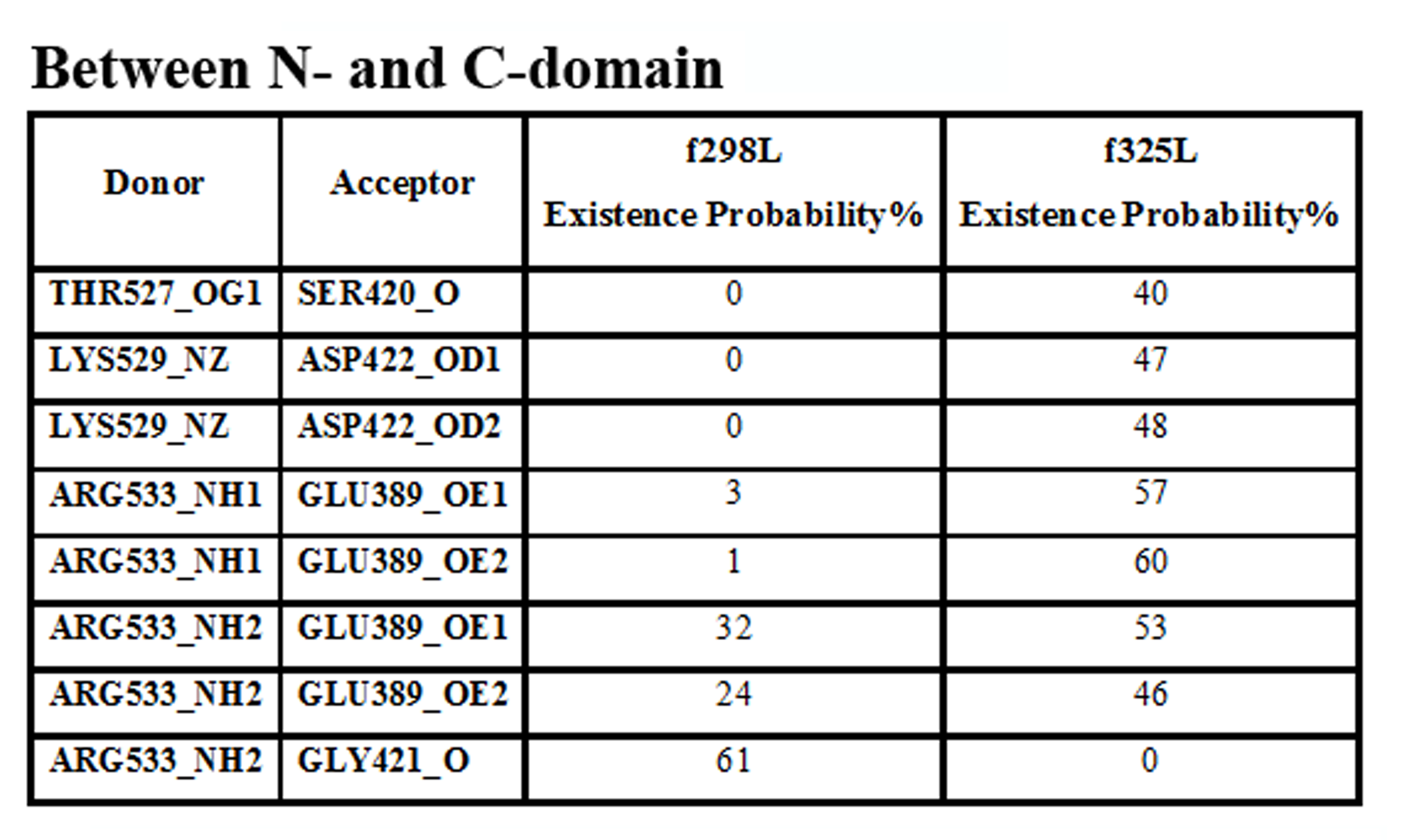

Supplement: S6 Table — (TIF) [file pone.0180667.s011.tif]
